# Research on Mechanical Fault Prediction Method Based on Multifeature Fusion of Vibration Sensing Data

**DOI:** 10.3390/s20010006

**Published:** 2019-12-18

**Authors:** Min Huang, Zhen Liu

**Affiliations:** Department of Software Engineering, South China University of Technology (SCUT), Guangzhou 510006, China; minh@scut.edu.cn

**Keywords:** vibration sensing data, fault prediction, artificial neural network, particle swarm optimization, Dempster-Shafer evidence theory

## Abstract

Vibration sensing data is an important resource for mechanical fault prediction, which is widely used in the industrial sector. Artificial neural networks (ANNs) are important tools for classifying vibration sensing data. However, their basic structures and hyperparameters must be manually adjusted, which results in the prediction accuracy easily falling into the local optimum. For data with high levels of uncertainty, it is difficult for an ANN to obtain correct prediction results. Therefore, we propose a multifeature fusion model based on Dempster-Shafer evidence theory combined with a particle swarm optimization algorithm and artificial neural network (PSO-ANN). The model first used the particle swarm optimization algorithm to optimize the structure and hyperparameters of the ANN, thereby improving its prediction accuracy. Then, the prediction error data of the multifeature fusion using a PSO-ANN is repredicted using multiple PSO-ANNs with different single feature training to obtain new prediction results. Finally, the Dempster-Shafer evidence theory was applied to the decision-level fusion of the new prediction results preprocessed with prediction accuracy and belief entropy, thus improving the model’s ability to process uncertain data. The experimental results indicated that compared to the K-nearest neighbor method, support vector machine, and long short-term memory neural networks, the proposed model can effectively improve the accuracy of fault prediction.

## 1. Introduction

Large-scale industrial machinery and equipment in the petroleum, chemical, aviation, and electricity sectors are the mainstays of modern economic development. Monitoring the operation status and fault prediction can effectively guarantee the safe and reliable operation of the equipment, which can result in huge economic benefits. Operational monitoring data obtained from mechanical equipment generally includes vibration signals, pressure, sound, and temperature. Among these parameters, the vibration signal contains a significant amount of useful information related to mechanical equipment [1,2] which can accurately reflect the operating state. At the same time, with the rapid development of communication technology and the improvement of computing capacity in recent years, the cost of vibration sensing data acquisition from mechanical equipment has been significantly reduced. Using multiple vibration sensors to obtain real-time operational data from different parts of the mechanical equipment, designing and selecting an appropriate data processing model [3] and accurately predicting mechanical equipment failure are critical to the development of intelligent mechanical equipment.

Presently, widely-used mechanical fault prediction methods employ artificial neural networks (ANNs) [4,5,6], support vector machines (SVMs) [7,8], deep learning [9,10,11], and other artificial intelligence (AI) technologies. For example, Ben et al. [12] proposed the use of empirical mode decomposition and energy entropy for feature extraction, which was combined with an ANN for multifeature fusion to make bearing fault predictions. Jiang et al. [13] used a variety of different time-domain analytical methods for feature extraction combined with SVM for multifeature fusion to achieve fault prediction for rotating machinery. Su et al. [14] proposed a new information fusion framework based on convolutional neural networks (CNNs), and residual squeeze networks were used to make fault predictions for high-speed trains. Yang et al. [15] proposed a time series analysis model based on a long short-term memory neural network to make fault predictions for electro-mechanical actuators. Dai et al. [16] proposed a multisource information fusion model based on a deep belief network to perform fault detection analyses on a power transformer. Jiang et al. [17] proposed a multifeature fusion method for stacked multilevel denoising autoencoders, which can effectively improve the fault diagnosis accuracy of wind turbines by using a deep network architecture formed by stacking.

Compared with SVM and deep learning algorithms, ANNs have lower requirements for training data, and they can quickly build a multifeature fusion model. However, the network structure and hyperparameters of the ANN must be manually adjusted, which causes the prediction accuracy to easily fall into the local optimum. To solve this problem, Illias et al. [18] proposed a hybrid modified evolutionary particle swarm optimization algorithm that optimizes the learning rate and momentum parameters of the ANN, but the number of hidden layer neurons had to be manually determined. Alnaqi et al. [19] proposed a hybrid particle swarm optimization algorithm to optimize the weight parameters and deviations of the ANN, but other parameters still required manual adjustment. Liao et al. [20] proposed a regrouping particle swarm optimization algorithm to optimize the weight parameters, deviations, and hidden layer neurons of the ANN, but there was no comparative analysis of learning parameters and other hyperparameters. Currently, many scholars use particle swarm optimization algorithms to optimize weight parameters [21], learning rate, and other hyperparameters [22] of the ANN, but they do not pay attention to the optimization of the hidden layer structure of the ANN, which results in the ANN training process still requiring the assistance of artificial experience.

Due to the complex operating environment associated with a great deal of mechanical equipment, vibration sensing data results in serious noise pollution and great uncertainty. For data with high levels of uncertainty, it is difficult for an ANN to provide accurate prediction results, which results in lower final prediction accuracies. Through investigation, Dempster-Shafer (DS) evidence theory was found to have a high decision-making ability for uncertain data. For example, Li et al. [23] proposed a bearing fault diagnosis model based on ensemble deep CNNs and improved DS evidence theory; the experimental results showed that it provided better diagnostic results than other machine learning methods. Kar et al. [24] proposed a multifeature fusion model based on an ANN and DS evidence theory, which can effectively improve the accuracy of fault prediction compared to the use of an ANN alone for bearing fault diagnosis. However, basic DS evidence theory has difficulty obtaining the correct decision results when the original evidence has high levels of conflict. To solve this problem, many types of belief entropy have been proposed to measure the uncertainty between different datasets. For example, Deng Yong [25] and Jiroušek et al. [26] proposed an improved belief entropy based on Shannon entropy, while Pan et al. [27] and Cui et al. [28] improved upon Deng entropy. According to belief entropy, scholars have proposed a variety of schemes to preprocess the original evidence, which is combined with DS evidence theory for information fusion. For example, Jiang et al. [29] and Tang et al. [30] used belief entropy to preprocess the original evidence. Wang et al. [31] proposed the preprocessing of original evidence using evidence distance and belief entropy. Xiao et al. [32] proposed the preprocessing of original evidence with improved cosine similarity and belief entropy; although this improved strategy combined with the DS evidence theory can be used in some scenarios, it is still unable to effectively improve the decision-making ability of the original DS evidence theory for uncertain data.

To address the above problems, the particle swarm optimization algorithm was used to optimize the ANN hyperparameters and hidden layer structure, which improved the prediction accuracy of the ANN. In addition, for data with false predictions based on the PSO-ANN multifeature fusion model, multiple PSO-ANN models trained with different single features were used for reprediction. At the same time, the prediction accuracy and belief entropy were applied to preprocess the new prediction results, which were combined with the DS evidence theory for the decision-level fusion of the preprocessed prediction results. The rest of the paper is organized in the following manner: Section 2 introduces a multifeature fusion model based on vibration sensing data. The feature extraction methods based on vibration sensing data are discussed in Section 3. The principle of applying a particle swarm optimization algorithm combined with an ANN for feature-level fusion is proposed in Section 4. Section 5 considers the principle of multiple PSO-ANN models using different single feature training combined with DS evidence theory for decision-level fusion. The selection of rolling bearings for multifeature fusion fault prediction experiments and the analytical results are presented in Section 6, and a brief summary is provided in Section 7.

## 2. Multifeature Fusion Model Based on Vibration Sensing Data

**Figure 1:**
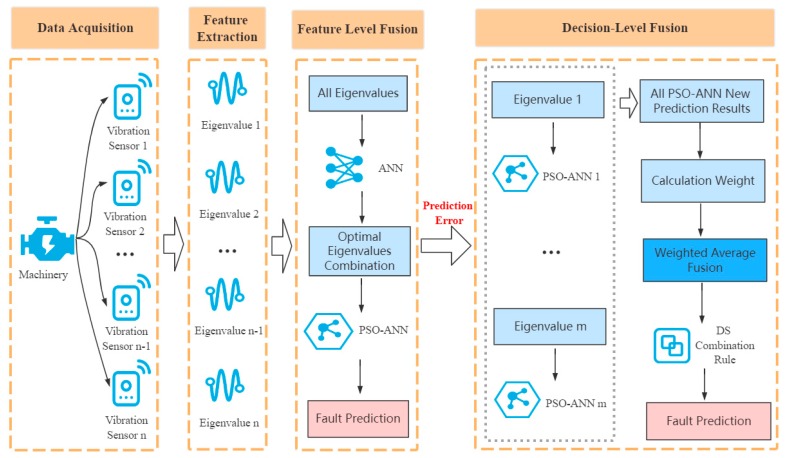
Multifeature fusion model based on vibration sensing data.

A vibration sensor can measure the impact force and acceleration of mechanical equipment, and it generally uses the acceleration data of the mechanical equipment to perform fault prediction. Various types of machinery, such as steam turbines, pumps, gearboxes, and machine tools, are composed of many components. With the increase in service life, each part can affect the vibration mode of the entire device. Different vibration modes may cause different faults. Through scientific analyses of the vibration signal, it is possible to effectively monitor the operating state of the mechanical equipment for better maintenance. Therefore, this paper proposes a multifeature fusion model based on vibration sensing data to analyze and process the vibration signals of mechanical equipment. The model was divided into four stages: data acquisition, feature extraction, feature-level fusion, and decision-level fusion. The details are presented in Figure 1.

**Stage 1: Data Collection**

As shown in the first stage in Figure 1, multiple vibration sensors are placed in different parts of the mechanical equipment that are prone to failure to collect real-time data.

**Stage 2: Feature Extraction**

As shown in the second stage in Figure 1, according to the selected sliding window size (the length of continuous time series of original vibration signals) and time-domain feature extraction method, the original vibration sensing data is extracted according to the time series sequence.

**Stage 3: Feature-Level Fusion**

As shown in the third stage in Figure 1, the ANN is first used to perform multifeature fusion on all the feature values extracted in the second stage, and the optimal feature combination is selected according to the prediction accuracy. Then, using the input data formed by the optimal feature combination, the PSO-ANN is used for feature-level fusion. Finally, the prediction error data of multifeature fusion using the PSO-ANN is repredicted using the decision-level fusion in the fourth stage.

**Stage 4: Decision-Level Fusion**

As shown in fourth stage in Figure 1, multiple PSO-ANN models using different single feature training first repredict the prediction error data to obtain new prediction results and fault prediction accuracies, and the weights of the corresponding model prediction results are calculated using the fault prediction accuracy and belief entropy. Then, weighted average fusion preprocessing is performed on the new prediction results using the weights. Finally, the DS evidence theory is used for the decision-level fusion of the preprocessed prediction results to obtain the final fault diagnosis results.

## 3. Feature Extraction Method Based on Vibration Sensing Data

Feature extraction can effectively reduce the uncertainty in vibration sensing data. Common feature extraction methods include information entropy [33,34,35], time domain analysis [36,37], empirical mode decomposition [38,39,40], and wavelet packet analysis [41,42]. Compared to the information entropy method and the empirical mode decomposition method, time domain analysis is less affected by the interruption of time-frequency signals, the steps of feature extraction are relatively simple, and different time domain features contain different information in the vibration signal. By comparing and analyzing the time domain feature extraction methods proposed in previous research, the latest or most widely-used feature extraction methods [43,44,45] were selected, as shown in Table 1.

The $x_{i}$ in all formulas in Table 1 represents vibration sensing data collected during the *i*-th unit time, and $\bar{x}$ represents the mathematical average of the vibration sensing data collected for *n* consecutive unit times (where n represents the sliding window size). The calculation and meaning of the parameter $W_{t-i}$ in the waveform entropy formula can be referred to in [45]. When using nine different time-domain feature extraction methods in Table 1 for feature extraction, the input vibration sensing data sequence and the sliding window size are the same.

## 4. Feature-Level Fusion Based on the Use of a PSO-ANN

This section introduces the process of applying the particle swarm optimization (PSO) algorithm combined with an ANN (PSO-ANN) for feature-level fusion, which is divided into three subsections. The structure of the ANN used in this study and the strategy to obtain the optimal combination of eigenvalues are introduced in Section 4.1. Section 4.2 introduces the optimization principle of the PSO algorithm combined with an ANN, and the algorithm principle of feature-level fusion using a PSO-ANN is discussed in Section 4.3.

### 4.1. Artificial Neural Network and the Strategy to Obtain the Optimal Eigenvalues Combination

**Figure 2:**
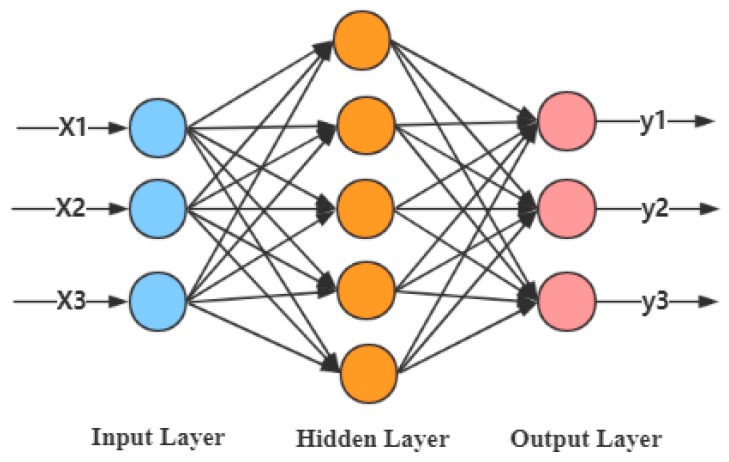
Structure of a three-layer ANN.

The basic structure of an ANN consists of an input layer, a hidden layer, and an output layer, with each layer containing a different number of neurons, as shown in Figure 2. During the training process, the learning rate, the number of hidden layer neurons, and the gradient descent algorithm must be set according to artificial experience. The commonly-used gradient descent algorithm includes stochastic gradient descent [46], momentum gradient descent [47], and the Adam optimization [48] algorithm, with the Adam optimization algorithm performing the best for practical applications. The momentum and RMSprop parameters are hyperparameters of the Adam optimization algorithm which must be manually adjusted during network training. The network structure and hyperparameter setting of the ANN are related to the artificial experience. If the setting is not ideal, it will result in a large labor cost, and it is easy to make the model prediction accuracy fall into the local optimum.

**Figure 3:**
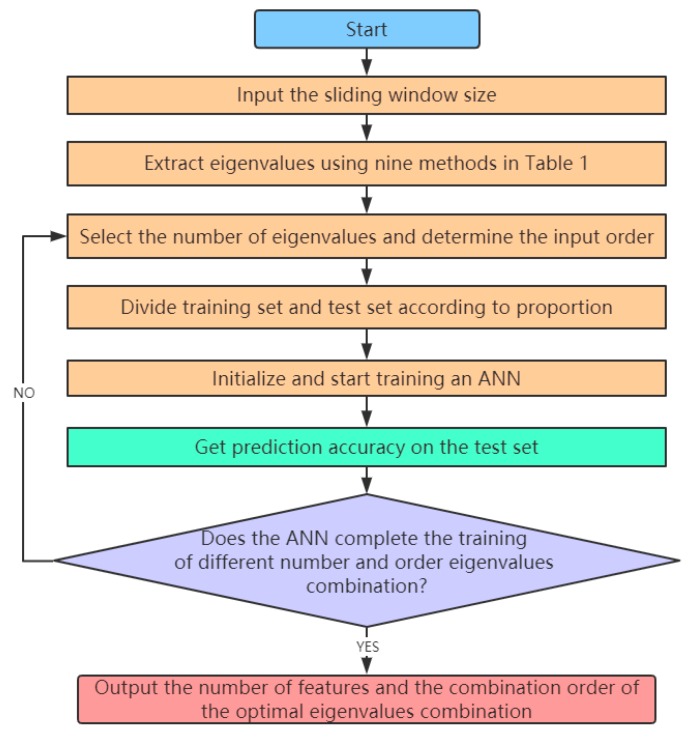
Using an ANN to get the optimal combination of eigenvalues.

In this study, the Adam optimization algorithm is selected as the gradient descent algorithm of the ANN. According to the feature-level fusion process in Figure 1, the strategy using an ANN to get the optimal combination of eigenvalues is shown in Figure 3. 

As shown in Figure 3, the number of eigenvalues and the combination order need to be continuously changed, and the combination of eigenvalues obtained each time are input to the ANN for training to get the prediction accuracy of the test set. Finally, the combination of eigenvalues with the highest prediction accuracy is the optimal combination of eigenvalues.

### 4.2. Optimization Principle Using the Particle Swarm Optimization Algorithm

The basic idea of the particle swarm optimization algorithm [49] is to initialize multiple random solutions of the problem to be optimized, with each solution corresponding to one particle, which is used to find the optimal solution in an N-dimensional space through cooperation and information sharing among multiple particles [50]. Each particle contains an N-dimensional velocity vector $V_{i}=\left(v_{i1},v_{i2},\ldots ,v_{in}\right)$ and a corresponding position vector $X_{i}=\left(x_{i1},x_{i2},\ldots ,x_{in}\right)$, where the velocity vector is used to adjust the motion path of the particle; the position vector represents a solution of the problem to be optimized. In this study, the prediction accuracy of the ANN was used as the problem to be optimized. The learning rate, the number of hidden layer neurons, the momentum parameter, and the RMSprop parameter were used to form the position vector of each particle. The particle swarm optimized the global position by iteration, and the update formula of the velocity vector and position vector of each particle can be expressed by Equations (1) and (2), respectively.

(1) $$\;V_{i}^{k+1}=wV_{i}^{k}+c_{1}r_{1}\left(Pbest_{i}^{k}-X_{i}^{k}\right)+c_{2}r_{2}\left(Gbest^{k}-X_{i}^{k}\right)$$

(2) $$\;X_{i}^{k+1}=X_{i}^{k}+V_{i}^{k+1}$$

In Equation (1), $Pbest_{i}^{k}$ represents the optimal position of the *i*-th particle in the *k*-th iteration, and $Gbest^{k}$ represents the optimal position of the particle swarm after *k* iterations. $r_{1}$ and $r_{2}$ are two random constants, and the range of values is [0, 1]. This is used to increase the randomness of the particle search, and w is the inertia weight parameter, which is used to adjust the range of the particle search for the current space [51]. The calculation formula is expressed as Equation (3).

(3) $$w=\;w_{max}-\left(w_{max}-w_{min}\right)\frac{iteration}{iteration_{max}}$$

where the value of $w_{max}$ is 0.9, and the value of $w_{min}$ is 0.4. iteration represents the current number of iterations, and $iteration_{max}$ represents the maximum number of iterations.

$c_{1}$ and $c_{2}$ are the acceleration learning constants used to adjust the maximum step size of the particle search [52]. The updated formula is expressed as Equation (4).

(4) $$c=\;c_{max}-\left(c_{max}-c_{min}\right)\frac{iteration}{iteration_{max}}$$

where the value of $c_{max}$ is 2.0, the value of $c_{min}$ is 0.5, and the values of $c_{1}$ and $c_{2}$ are equal to c.

### 4.3. Algorithm Principle of Feature-Level Fusion Using a PSO-ANN

In this study, the PSO algorithm was used to optimize the learning rate, the number of hidden layer neurons, the momentum parameter, and the RMSprop parameter of the ANN, which avoids the process of manual parameter adjustment and realizes the automatic optimization of the network structure and parameters. When a particle swarm is used to optimize the position iteratively, the current position must be determined based on the fitness value obtained by the fitness function. In this study, the loss error generated by the ANN during the training process was selected, and the prediction accuracy of the test set was used as the return value of the fitness function. The cross-entropy [53] was used to calculate the loss error of ANN training. The calculation formula is expressed as Equation (5).

(5) $$\mathrm{Loss}=\;-\frac{1}{n}\overset{n}{\underset{i=1}{\sum }}\left(y_{i}\log{\hat{y}}_{i}+\left(1-y_{i}\right)\log\left(1-{\hat{y}}_{i}\right)\right)$$

**Figure 4:**
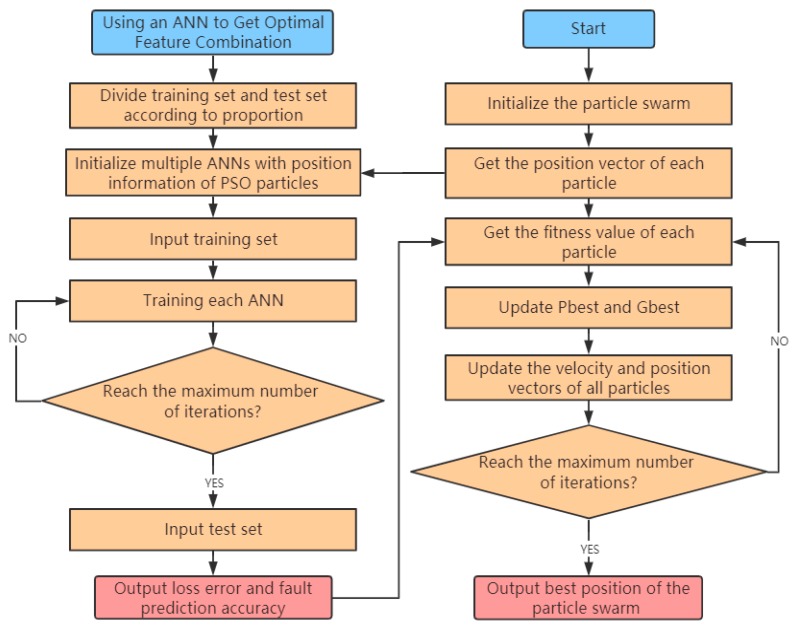
Flow chart of feature-level fusion using the PSO-ANN model.

The flow chart of feature-level fusion using the PSO-ANN model is shown in Figure 4, and the specific implementation process is presented as Algorithm 1.

In Algorithm 1, lines 11–13 indicate that the ANN is initialized by the position vector of particles, and the ANN is trained by the training set. Lines 14–15 represent the loss error of the ANN on the training set, and the prediction accuracy on the test set was used as the fitness value of the particle. Lines 16–23 indicate that the best position Pbest of the current particle and the best position Gbest of the particle swarm are updated based on the fitness value. Lines 24–27 indicate that the velocity vector and position vector of the current particle are updated according to Equations (1) and (2), respectively.

**Algorithm 1: **PSO-ANN algorithm.**Input:** All the eigenvalues of the optimal feature combination.**Output:** The best position of the particle swarm Gbest, and the best prediction accuracy.01: Set the parameters {n,$\;iteration_{max}$, $v_{max}$, $v_{min}$, $x_{max}$, $x_{min}$} 02: **for** i = 1 to n **do ** /* n is the number of particles */ 03:        Initialize $v_{i}$ = ($v_{i1},\;v_{i2},\;v_{i3},\;v_{i4}$), $x_{i}$ = ($x_{i1},\;x_{i2},\;x_{i3},\;x_{i4}$), $Pbest_{i}=\;x_{i}$ 04: **end for** 05: Acquire training set $X_{train}$, $Y_{train}\;$and test set $X_{test}$, $Y_{test}$ 06: Set the particle with best $\mathrm{fitness}\left(Pbest_{i}\right)$ to be Gbest 07: **for **k = 1 $\mathrm{to}\;iteration_{max}\;$**do**
08:        Update w with Equation (3) 09:        Update $c_{1}$, $c_{2}$ with Equation (4) 10:        **for** i = 1 to n **do** 11:                $ann_{i}=$ ann_model(learning_rate = $x_{i1}$, hidden_layer_ neurons = $x_{i2}$, 12:                                momentum_parameter = $x_{i3}$, rmsprop_parameter = $x_{i4}$) 13:                $ann_{i}$.fit($X_{train}$, $Y_{train}$) /* Training ANN model */ 14:                $loss\_value_{i}$ = $ann_{i}$.loss_value 15:                $prediction\_accuracy_{i}$ = $ann_{i}$.score($X_{test}$, $Y_{test}$) 16:                **if** ($loss\_value_{i}$ > fitness($Pbest_{i}$).loss_value and 17:                        $prediction\_accuracy_{i}$ < fitness($Pbest_{i}$).prediction_accuracy) **then**
18:                    $Pbest_{i}=\;x_{i}$ 19:                **end if** 20:                ** if** ($loss\_value_{i}$ > fitness(Gbest).loss_value and 21:                        $prediction\_accuracy_{i}$ < fitness(Gbest).prediction_accuracy) **then**
22:                    $\mathrm{Gbest}=\;x_{i}$ 23:                **end if** 24:                **for** j = 1 to 4 **do**
25:                        $\;v_{ij}^{k+1}=wv_{ij}^{k}+c_{1}r_{1}\left(Pbest_{ij}^{k}-x_{ij}^{k}\right)+c_{2}r_{2}\left(Gbest_{j}^{k}-x_{ij}^{k}\right)$
26:                        $\;x_{ij}^{k+1}=x_{ij}^{k}+v_{ij}^{k+1}$
27:                **end for**
28:        **end for** 29: **end for**

## 5. Decision-Level Fusion Based on Multiple PSO-ANN Models and Dempster-Shafer Evidence Theory

This section introduces the principle of decision-level fusion of multiple PSO-ANN models with different single feature training combined with DS evidence theory (PSO-ANN-DS), which is divided into two subsections. In Section 5.1, the running process of decision-level fusion using four PSO-ANN models with different single feature training combined with DS evidence theory is introduced. The principle of preprocessing new prediction results using prediction accuracy and belief entropy, and the algorithm model of decision-level fusion combined with DS evidence theory, are discussed in Section 5.2.

### 5.1. Running Process of a PSO-ANN-DS

The nine time domain feature extraction methods presented in Table 1 can extract feature information from different aspects of the vibration sensing data. For most vibration sensing data, multifeature fusion can effectively improve the accuracy of mechanical fault prediction. However, the noise pollution of partial vibration sensing data is serious; the reasons for the noise in the vibration sensing data of mechanical equipment are shown in Table 2.

**Figure 5:**
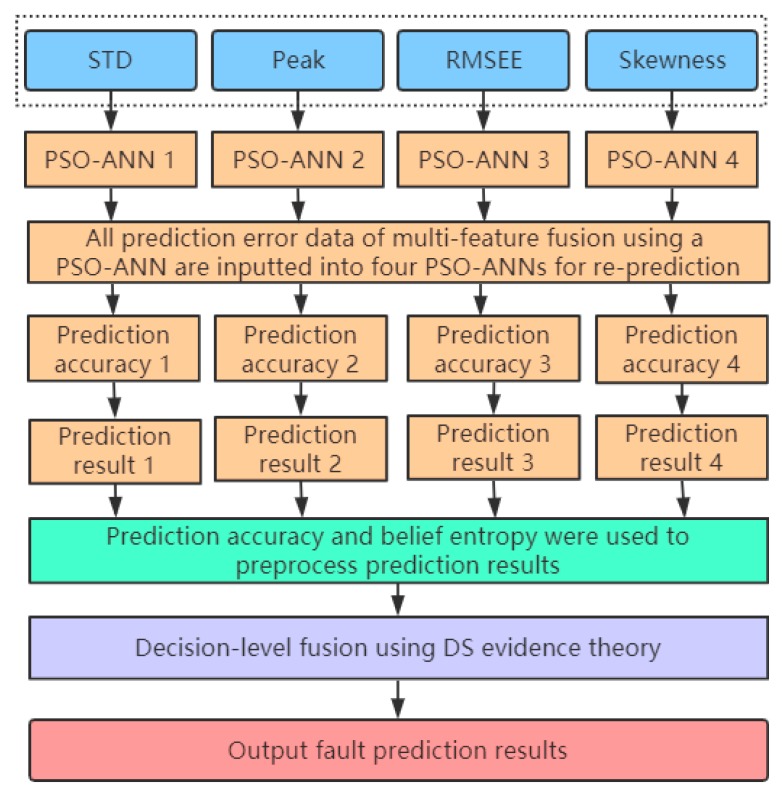
Running flowchart of decision-level fusion using a PSO-ANN-DS model.

As shown in Table 2, the noise pollution of the vibration sensing data comes from mechanical equipment and vibration sensors; some of the reasons for this are difficult to control during the data acquisition process. Due to the serious noise pollution of the partial vibration sensing data, the uncertainty of the eigenvalue obtained by the partial time domain feature extraction method is relatively large. Therefore, it is difficult to obtain accurate prediction results by using the PSO-ANN model for multifeature fusion. For data containing a significant amount of noise pollution, the prediction results of multiple PSO-ANN models trained with partially different single features are less uncertain than the multifeature fusion using a PSO-ANN model. However, multiple PSO-ANN models trained with different single features may have different prediction results, and the final results are still difficult to determine. Through investigation, the Dempster-Shafer (DS) evidence theory can effectively integrate multiple uncertain prediction results [54], and it is widely used in the field of information fusion [55]. Therefore, this paper uses four PSO-ANN models trained by different single features (STD, Peak, RMSEE and Skewness) to repredict the vibration sensing data with serious noise pollution. At the same time, the DS evidence theory is applied to the decision-level fusion of the new prediction results to obtain the final fault prediction results. The running process of the PSO-ANN-DS model is shown in Figure 5.

### 5.2. Algorithm Principle of Decision-Level Fusion Using a PSO-ANN-DS

**Figure 6:**
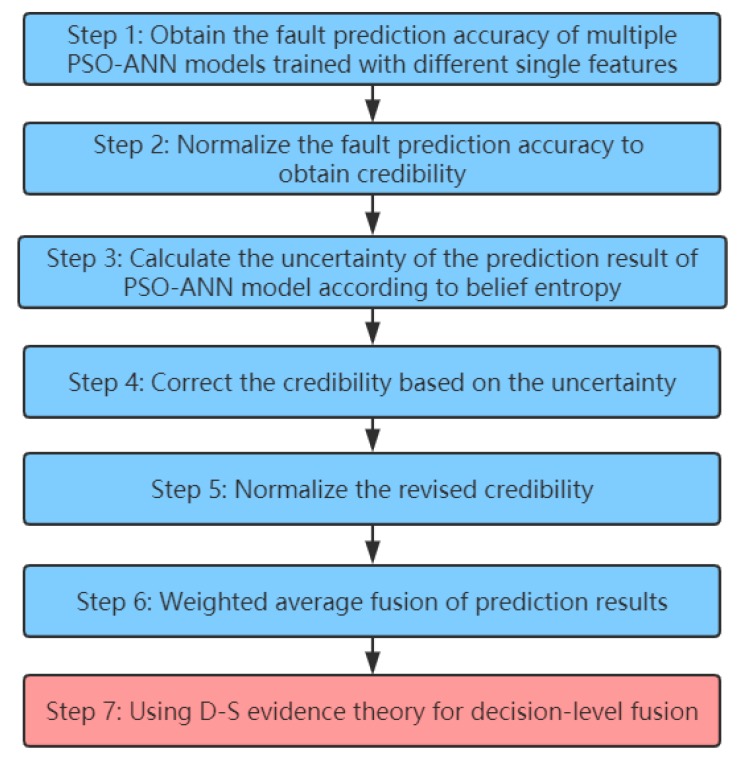
Flow chart of algorithm for decision-level fusion based on multiple PSO-ANN models combined with DS evidence theory.

As shown in Figure 5, four PSO-ANN models are first trained by different single features (STD, Peak, RMSEE, and Skewness). Then, all prediction error data of multifeature fusion using a PSO-ANN are inputted into four PSO-ANN models for reprediction, and the prediction results and prediction accuracies are obtained. However, it is difficult to directly obtain correct decision results using the DS evidence theory for results with large conflicts. Therefore, the prediction accuracy of multiple PSO-ANN models trained by different single features and belief entropies are used to perform weighted average fusion preprocessing of the prediction results, which is then combined with the DS evidence theory for decision-level fusion. The algorithm flow of decision-level fusion using multiple PSO-ANN models trained with different single features combined with the DS evidence theory is shown in Figure 6.

Step 1: Obtain the fault prediction accuracy of multiple PSO-ANN models trained with different single features, which is recorded as PRE.

(6) $$\;\mathrm{PRE}=\left\{{\mathrm{Pre}}_{1},{\;\mathrm{Pre}}_{2},\ldots ,{\mathrm{Pre}}_{i},\ldots ,{\mathrm{Pre}}_{n}\right\}$$

where ${\mathrm{Pre}}_{i}$ represents the fault prediction accuracy of the PSO-ANN model trained with the ith single eigenvalue of the test set with a high level of uncertainty. In this paper, the value of n is 4, and different PSO-ANN models are trained by STD, Peak, RMSEE, and Skewness.

Step 2: Normalize the fault prediction accuracy to obtain credibility, recorded as CRD.

The PRE is normalized to obtain the credibility of each PSO-ANN model.

(7) $$\mathrm{CRD}\left(m_{i}\right)=\;\frac{{\mathrm{Pre}}_{i}}{\sum_{i=1}^{n}{\mathrm{Pre}}_{i}}$$

where $m_{i}$ is the fault prediction result of the PSO-ANN model trained with a single feature *i*.

Step 3: Calculate the uncertainty of fault prediction result of PSO-ANN model according to belief entropy, recorded as MUN.

Belief entropy is an important indicator used to measure uncertainty; the greater the value of belief entropy, the greater the uncertainty in the information. Many scholars have proposed a specific belief entropy based on DS evidence theory; Deng entropy [25] is the most widely used. The calculation formula is expressed as Equation (8).

(8) $$E\left(m\right)=-\underset{A\in 2^{\theta }}{\sum }m\left(A\right)\log\left[\frac{m\left(A\right)}{2^{\left|A\right|}-1}\right]$$

To avoid the occurrence of 0 in the belief entropy calculation result, specific mathematical processing is performed using Equation (9).

(9) $$\mathrm{MUN}\left(m_{i}\right)={\;e}^{E\left(m_{i}\right)}\;$$

Step 4: Correct the credibility based on the uncertainty, which is recorded as MCRD.

(10) $$\mathrm{MCRD}\left(m_{i}\right)=\;\mathrm{CRD}\left(m_{i}\right)*\;\mathrm{MUN}\left(m_{i}\right)$$

Step 5: Normalize the revised credibility, recorded as NMCRD.

(11) $$\mathrm{NMCRD}\left(m_{i}\right)=\;\frac{\mathrm{MCRD}\left(m_{i}\right)}{\sum_{i=1}^{n}\mathrm{MCRD}\left(m_{i}\right)}$$

Step 6: Weighted average fusion of prediction results, recorded as WAE.

(12) $$\mathrm{WAE}\left(m\right)=\sum_{i=1}^{n}\left(\mathrm{NMCRD}\left(m_{i}\right){*m}_{i}\right)$$

Step 7: Using DS evidence theory for decision-level fusion.

It is assumed that $m_{1}$ and $m_{2}$ are the PSO-ANN model fault prediction results trained by feature 1 and feature 2, respectively, where A, B, and C represent the fault type. Then, the final decision result m (C) obtained using the Dempster-Shafer synthesis rule is expressed as Equation (13).

(13) $$m\left(C\;\right)=m_{1}⊕m_{2}=\;\{\begin{array}{c} \frac{1}{1-K}\sum_{A\cap B=C}m_{1}\left(A\right)m_{2}\left(B\right),\;C\;\neq \;\emptyset \\ \;0,\;C\;=\;\emptyset \end{array}$$

*K* represents the collision coefficient, and *K* < 1, which is defined as follows:

(14) $$K=\;\sum_{A\cap B=\emptyset }m_{1}\left(A\right)m_{2}\left(B\right)$$

According to the literature [56], the original DS evidence synthesis rule is used to continuously fuse the WAE(m) *n* − 1 times, where n represents the total number of different single features. The formula is expressed as Equation (15).

(15) $$\mathrm{Fus}\left(m\right)=({\left({\left(\mathrm{WAE}\left(m\right)⊕\mathrm{WAE}\left(m\right)\right)}_{1}⊕\ldots {)}_{h}\mathrm{WAE}\left(m\right)\right)}_{\left(n-1\right)}$$

Fus(m) is the final decision result, and the specific implementation process is presented as Algorithm 2.

**Algorithm 2: **PSO-ANN-DS algorithm.**Input: **Four single eigenvalues, and fault data with high levels of uncertainty.**Output: **Decision-level fusion result Fus(m).01: /* Step 1 */ 02: Train_data = {STD, Peak, RMSEE, Skewness} /* Four single eigenvalues */ 03: **for** i = 1 to 4 **do**
04:        $PSOANN_{i}$ = PSO-ANN_algorithm(Input = Train_data [i]) 05:        PRE[i] = $PSOANN_{i}$(test_data = fault data with high 06:                                uncertainty). prediction_accuracy 07: **end for** 08: /* Step 2 */ 09: **for** i = 1 to 4 **do**
10:        CRD[i] = PRE[i] / sum(PRE) 11: **end for** 12: /* Step 3 */ 13: **for** i = 1 to 4 **do**
14:        MUN[i] = Calculate the value with Equation (8) and (9) 15: **end for** 16: /* Step 4 */ 17: **for** i = 1 to 4 **do** 18:        MCRD[i] = CRD[i] * MUN[i] 19: **end for** 20: /* Step 5 */ 21: **for** i = 1 to 4 **do**
22:        NMCRD[i] = MCRD[i] / sum(MCRD) 23: **end for** 24: /* Step 6 */ 25: **for** j = 1 to J **do** /* J is the number of fault types */ 26:        WAE[j] = 0 27:        ** for** i = 1 to 4 **do**
28:                WAE[j] = WAE[j] + NMCRD[i] * $PSOANN_{i}$.prediction_result(fault_type = j) 29:        **end for** 30: **end for** 31: /* Step 7 */ 32: Fus(m) = WAE 33: **for** i = 1 to 3 **do** /* There are 4 single features, which need to be merged 3 times. */ 34:        Fus(m) = Fus(m) WAE /* refers to the DS fusion rule */ 35: ****end for****

## 6. Bearing Fault Prediction Experiment Based on Vibration Sensing Data

This section describes the bearing failure prediction experiment based on vibration sensing data, which is divided into five subsections. Section 6.1 introduces the data set and experimental environment used in this experiment. The application of the ANN for multifeature fusion fault diagnosis and the means of obtaining the optimal feature combination according to the prediction accuracy are introduced in Section 6.2. The input data obtained according to the optimal feature combination and the use of PSO to optimize the structure and parameters of the ANN are described in Section 6.3. The data used for feature-level fusion prediction errors using the PSO-ANN model are presented in Section 6.3, and the use of the PSO-ANN-DS model for decision-level fusion to improve the accuracy of PSO-ANN fault prediction is introduced in Section 6.4. Finally, in Section 6.5, the fault prediction accuracies of various models are compared and analyzed.

### 6.1. Introduction to Data Set and Experimental Environment

This paper uses the bearing fault data set [57] provided by Case Western Reserve University (CWRU) as the experimental data source. CWRU’s laboratory used bearing motors for experiments to collect vibration data using accelerometers near and away from the bearing. The bearing used in the experiment was artificially damaged by electric sparks, and the failure parts included the inner ring, the outer ring, and the ball at the drive end or the fan end of the bearing. The balls were recorded as different types of faults according to different diameters. There were four different ball diameters: 0.007, 0.014, 0.021, and 0.028 inches. The outer ring used an accelerometer to collect data in the fault areas at 3:00, 6:00, and 12:00. In addition, there were four types of motor load used in the experiments: 0HP, 1HP, 2HP, and 3HP. There were also four types of rotational speeds: 1797 rpm, 1772 rpm, 1750 rpm, and 1730 rpm. In this paper, some data was selected from the CWRU data set for the experiment. The specific data is presented in Table 3.

**Figure 7:**
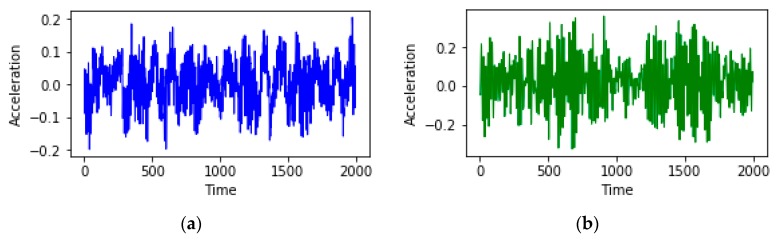
The change of acceleration value with a continuous unit time under (**a**) normal state and (**b**) rolling element fault conditions.

As shown in Table 3, there are six types of mechanical faults, namely normal state, inner raceway fault, rolling element fault (Ball), outer race orthogonal@3:00 fault, outer race centered@6:00 fault, and outer race opposite@12:00 fault. The vibration sensing data of bearing motors collected in 2000 consecutive unit times for two fault types (normal state and rolling element fault) are selected, and the change of acceleration value with a continuous unit time is shown in Figure 7.

As shown in Figure 7, there is a clear difference between the vibration sensing data of the bearing motors under normal state and rolling element fault conditions. In addition, the acceleration value shows a trend of periodic changes with the increase of unit time.

An Ubuntu 18.04 operating system with 32 G of memory and an Intel i7-8700k CPU were the important components of our experimental computer. Python was used as the basic development language, and the ANN was implemented by Sklearn.

### 6.2. Using an ANN to Get Optimal Feature Combination

**Figure 8:**
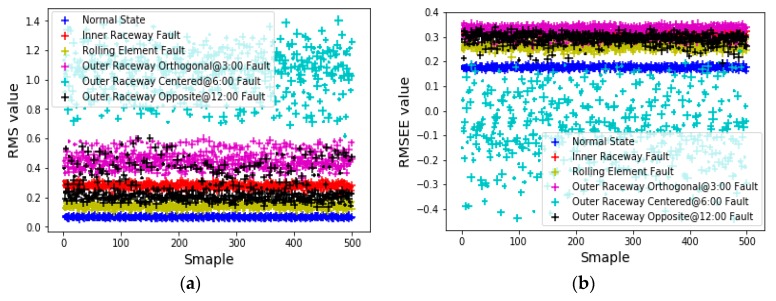
Sample points distribution using (**a**) RMS and (**b**) RMSEE formulas for feature extraction.

The setting of the sliding window size not only has a great influence on the optimal feature combination, but also has a great influence on the final prediction accuracy. To compare the effects of different sliding window values on the accuracy of final fault prediction, sliding window sizes of 120, 240, 360, 480, 600, 720, 840, and 960 were employed for feature value extraction. Each type of fault data was extracted into 500 groups, and a total of 3000 groups (six different types) were extracted. For example, 500 groups of sample points were each extracted according to the RMS formula and RMSEE formula when the sliding window size was set to 840; the distribution of six different mechanical fault feature sample points is shown in Figure 8.

According to the artificial experience of the ANN model parameters and structure adjustment, the number of ANN hidden layers used in this experiment was set to 1, the number of hidden layer units was set to 20, the learning rate was set to 0.001, the momentum parameter value was set to 0.9, and the RMSprop parameter value was set to 0.999. The training data set used in the experiment accounted for 70% of the total data, while the test set accounted for the remaining 30%. Table 4 shows the accuracy of the ANN for single feature fault prediction and multifeature fusion fault prediction using different sliding window sizes for feature value extraction.

As shown in Table 4, as the size of the sliding window was increased, the fault accuracy using multifeature fusion exhibited an increasing trend. However, when the size of the sliding window was increased from 840 to 960, the accuracy of multifeature fusion fault prediction only slightly improved, and the accuracy of partial single feature failure prediction decreased. Based on the results of additional experimental comparisons, when the sliding window size continued to increase to 960, the final fault diagnosis prediction accuracy was virtually unchanged.

When using an ANN for multifeature fusion fault prediction, the number or order of eigenvalue combinations have a greater impact on the accuracy of fault prediction. The sliding window size was set to 360, 2–9 different features were applied in turn to merge, and the order of feature fusion was changed. The prediction accuracy is shown in Table 5.

As shown in Table 5, when multifeature fusion is performed in the order of RMS, STD, Peak, RMSEE, WFE, Kurtosis, Skewness, and CRF, the accuracy of fault prediction is the highest. By comparing the last column and the first three columns of Table 5, the accuracy of the fault prediction using nine features was found to be much higher than that associated with the application of a few features. In addition, as shown in the last column of Table 5, the fault prediction accuracy of the same number of feature value combinations using Skewness or CRF to initiate multifeature fusion was the lowest, which was 1.33% lower than the highest accuracy. In accordance with the experimental strategy used in Table 5, the sliding window size was set to other values in turn, and the corresponding optimal feature combination and fault prediction accuracy were obtained. The results are shown in Table 6.

As shown in Table 6, when the sliding window values were set to 120, 360, and 720, there were eight optimal feature combinations. When the sliding window values were set to 240, 480, 600, 840, and 960, there were nine optimal feature combinations. For different sliding windows, the order of feature combinations may also be different.

### 6.3. Feature-Level Fusion Fault Prediction Experiment Based on a PSO-ANN

In this subsection, the input data formed by the optimal feature combination in Table 6 were used, and the hidden layer structure and hyperparameters of the ANN were automatically optimized using a PSO algorithm to avoid the process of manually adjusting the structure and parameters of the ANN model. The information in the parameter range of the ANN optimized by a PSO algorithm is shown in Table 7.

**Figure 9:**
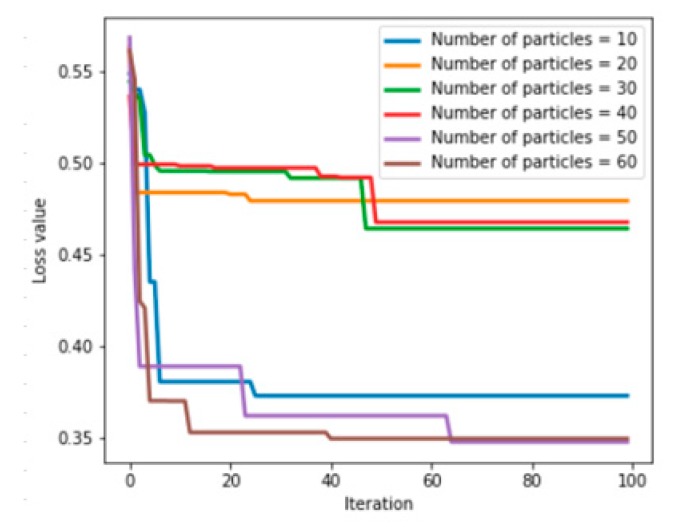
Relationship between the number of PSO iterations and the ANN’s loss value.

The application of a PSO algorithm also requires relevant parameters to be set. In addition to the inertia parameters and acceleration learning constants, the number of particles is also important. The larger the number of particles, the larger the search range of the a PSO algorithm, which leads to an increase in computational cost. If there are a small number of particles, the search range of the PSO algorithm is small, which makes it difficult to obtain solutions that meet the expected goals. The eigenvalues extracted when the sliding window size is 360 are used as the input data of the PSO-ANN model, and different particle swarm numbers (10, 20, 30, 40, 50, and 60, respectively) are used to initialize the PSO-ANN model for multifeature fusion fault prediction. The relationship between the number of iterations of the PSO algorithm initialized by different numbers of particles and the loss value of the ANN is shown in Figure 9 (the maximum number of iterations of the PSO algorithm was uniformly set to 100). Table 8 shows the parameter values, the loss values, and the prediction accuracy obtained by multifeature fusion fault prediction using the PSO-ANN model initialized with a different number of particles.

**Figure 10:**
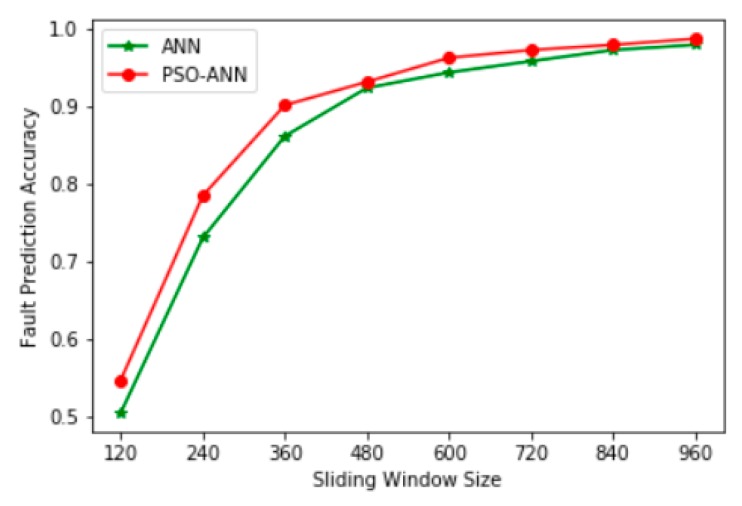
The relationship between the accuracy of fault prediction and the size of sliding window using multifeature fusion with the ANN and the PSO-ANN.

As shown in Figure 9 and Table 8, when the number of particles was set to 50, the PSO-ANN model achieved the highest prediction accuracy and the loss value was also the lowest. Therefore, when the number of particles is set to 50, the large calculation cost caused by the high number of particles is avoided and a better prediction accuracy is obtained. Table 9 shows the eigenvalues extracted using other sliding window sizes as training data and the accuracy of fault prediction using the PSO-ANN model, in which the number of particles is uniformly set to 50. Figure 10 shows the relationship between the fault prediction accuracy and the sliding window size using multifeature fusion with the ANN and the PSO-ANN.

A comparison of the prediction accuracy of single eigenvalues in Table 4 and Table 9 indicated that using the PSO to automatically optimize the ANN’s number of hidden layer neurons, learning rate, momentum parameter, and RMSprop parameter can effectively improve its prediction accuracy and avoid the process of manually adjusting the structure and parameters of the ANN model. In addition, as shown in Figure 10, the PSO-ANN model had a higher prediction accuracy than the ANN model for multifeature fusion fault diagnosis.

### 6.4. Decision-Level Fusion Fault Prediction Experiment Based on PSO-ANN-DS

In this subsection, the multifeature fusion fault prediction data from the PSO-ANN model were input into multiple PSO-ANN models trained by different single features for reprediction. It was then combined with DS evidence theory for decision-level fusion. Table 10 shows the reprediction results of multiple PSO-ANN models trained with different single eigenvalues using one of the prediction error data (the sliding window size of the eigenvalue extraction is 120). Table 11 shows the values of various parameters obtained by preprocessing the data in Table 10 and applying the prediction accuracy and belief entropy according to Algorithm 2.

The prediction results of the plurality of the PSO-ANN models in Table 10 were preprocessed according to the parameter values calculated in Table 11. The fusion was performed three times in combination with the DS evidence theory, and the fusion results are shown in Table 12.

According to Table 12, after three consecutive fusions, the maximum probability of outer race centered@6:00 fault is 0.36. It can be seen that the final fault prediction result is outer race centered@6:00 fault, which is the same as the real value. Based on the results of the PSO-ANN multifeature fusion fault prediction experiment in Section 6.3, Table 13 shows that multiple PSO-ANN models trained with different single features are combined with basic DS evidence theory, DS evidence theory and Deng entropy [30], DS evidence theory combined with evidence distance and Deng entropy [31], DS evidence theory combined with cosine similarity and Deng entropy [32], and the proposed method for fault prediction accuracy of decision-level fusion.

As shown in Table 13, the method proposed in [30] had a prediction accuracy that was lower than that of the basic DS theory when the sliding window sizes were 120 and 960. When the sliding window sizes were 240 and 360, the prediction accuracies of the methods proposed in [31,32] were also lower than those of the basic DS evidence theory. Compared with the basic DS evidence theory results, the method proposed and employed in this study could be used to effectively guarantee the original prediction accuracy and achieve different degrees of improvement.

### 6.5. Comparison and Analysis of Fault Prediction Accuracy of Various Models

In Section 6.2, according to the artificial experience, the ANN with a fixed structure and parameters was used for multifeature fusion fault prediction. The effects of different sliding window sizes on the prediction accuracy and optimal feature combinations were compared and analyzed. The experimental results revealed that when the sliding window size was less than 960, increasing the sliding window size could effectively improve the prediction accuracy of the ANN. By comparing and analyzing the optimal feature combination results corresponding to different sliding window sizes, the number and order of feature combinations will have a greater impact on the prediction accuracy of ANN. It is generally difficult to obtain the ideal prediction accuracy by manually adjusting the structure and hyperparameters of the ANN, while it is relatively easy to make the prediction accuracy fall into a local optimum. To solve this problem, in Section 6.3, PSO was used to automatically optimize the number of hidden layers, learning rate, momentum parameter, and RMSprop parameter of the ANN according to the input data formed by the optimal feature combination. The experimental results indicated that the prediction accuracy of the PSO-ANN model was significantly higher than that of the ANN. Because there are uncertain data in the original vibration sensing data, it is easy to generate a large deviation for the multifeature fusion using the PSO-ANN for this part of the data. Therefore, in Section 6.4, multiple PSO-ANN models trained with different single features were combined with the DS evidence theory for a decision-level fusion of uncertain data, thus improving the processing ability of the model for the uncertain data.

To further compare the fault prediction accuracy of the PSO-ANN-DS model proposed in this study with the accuracy of other models, the fault prediction accuracy based on the k-nearest neighbor (KNN) method, decision tree, random forest, naive Bayes, ANN, support vector machine (SVM), long- and short-term memory neural network (LSTM), PSO-ANN, and PSO-ANN-DS models using different sliding windows for feature extraction fault prediction accuracy are presented in Table 14.

As shown in Table 14, the ANN performed poorly in many models, while SVM and random forest had significant advantages over traditional classification methods. When the sliding window size was larger than 240, the prediction accuracy of the PSO-ANN was higher than the SVM prediction accuracy, but it was lower than that of random forest. Compared with the KNN method, SVM, and LSTM, the PSO-ANN-DS model had a significant advantage in fault prediction accuracy.

## 7. Conclusions

The multifeature fusion fault prediction method based on vibration sensing data is currently a hot research topic and a primary focus of future industrial development. The basic structure and hyperparameters of the ANN generally require manual adjustment, and it is easy to make the prediction accuracy fall into a local optimum. Therefore, based on the training data formed by the optimal feature combination, the PSO was used to optimize the number of hidden layers, learning rate, momentum parameters, and RMSprop parameters of the ANN to avoid the process of manual adjustment. The experimental results indicated that the prediction accuracy of the PSO-ANN was significantly higher than that of the ANN. The original vibration sensing data included data with serious noise pollution and a high degree of uncertainty, which led to incorrect results when the PSO-ANN model was applied for multifeature fusion fault prediction. For this part of the data, the PSO-ANN model’s prediction accuracy and belief entropy were used to preprocess the new prediction results, which were then combined the DS evidence theory for decision-level fusion. The experimental results revealed that compared with the original DS evidence theory or the combination of belief entropy, the proposed method can effectively improve the model’s ability to deal with uncertain data. In addition, compared to other models such as the KNN method, SVM, and LSTM, using the PSO-ANN-DS model for fault diagnosis resulted in high level of fault prediction accuracy.

## Figures and Tables

**Table 1:** Nine different time domain feature extraction methods based on vibration sensing data.

Serial Number	Feature Name	Formula
1	Root mean square (RMS)	$\sqrt{\frac{1}{n}\sum_{i=1}^{n}{\left(x_{i}\right)}^{2}}$
2	Standard deviation (STD)	$\sqrt{\frac{1}{n}\sum_{i=1}^{n}{\left(x_{i}-\bar{x}\right)}^{2}}$
3	Peak	max(x)
4	Root mean square entropy estimator (RMSEE)	$\frac{1}{n}\sum_{i=1}^{n}\left(\;-RMS\left(i\right)*\log\left(RMS\left(i\right)\right)\;\right)$
5	Waveform entropy (WFE)	$\frac{1}{M}\sum_{i=1}^{M}\left(\;W_{t-i}*\log\left(W_{t-i}\right)\;\right)$
6	Kurtosis	$\frac{1}{n-{\left(STD\right)}^{4}}\sum_{i=1}^{n}{\left(x_{i}-\bar{x}\right)}^{4}$
7	Skewness	$\frac{1}{n}\sum_{i=1}^{n}{\left(\frac{x_{i}-\bar{x}}{STD}\right)}^{3}$
8	Crest factor (CRF)	$\frac{Peak}{RMS}$
9	Impulse factor (IMF)	$\frac{Peak}{\frac{1}{n}\sum_{i=1}^{n}\left|x_{i}\right|}$

**Table 2:** Causes of vibration sensing data noise pollution.

Noise Location	Reason	Explanation
Mechanical equipment	Eddy noise	Increased external air velocity causes eddies around machinery.
	Rotating noise	The vibration force of rotating machinery deviates easily from the normal value when encountering strong air flow.
	Energy shortage	Energy issues (for example, oil level below average) cause large levels of noise pollution.
	Impact noise	Large levels of noise pollution caused by impacts.
	Other reasons	Suddenly increasing the operating power of mechanical equipment, manual operation of mechanical equipment.
Vibration sensor	Temperature factor	In general, the higher the temperature, the greater the measurement error.
	Resonant frequency	The closer the vibration frequency of the machine is to the value of the resonance frequency, the greater the measurement error.
	Placement deviation	Vibration sensors generally get acceleration sensing data in three directions. The larger the deviation in the placement direction, the greater the measurement error.
	Original error	Different types of vibration sensors have different original errors.
	Other environmental factors	Under the condition of a strong electrostatic field, alternating magnetic field, or nuclear radiation, the measurement error may become larger.

**Table 3:** Data used in the experiments in this study. The motor load was 1HP, the speed was 1772 rpm, and the ball diameter of the fault data was 0.007 inches.

Fault Type	File Name
Normal Baseline Data	98.mat
48K Drive End Bearing Fault Data (Inner Race)	110.mat
48K Drive End Bearing Fault Data (Ball)	123.mat
48K Drive End Bearing Fault Data (Outer Race Orthogonal@3:00)	149.mat
48K Drive End Bearing Fault Data (Outer Race Centered@6:00)	136.mat
48K Drive End Bearing Fault Data (Outer Race Opposite@12:00)	162.mat

**Table 4:** Fault prediction accuracy of feature value extraction using different sliding windows, where “All” is the fault accuracy of multifeature fusion according to the order of RMS, STD, Peak, RMSEE, WFE, Kurtosis, Skewness, CRF, and IMF.

Eigenvalue	Sliding Window Size							
	120	240	360	480	600	720	840	960
RMS	31.67%	52.11%	77.22%	86.11%	88.67%	88.11%	91.33%	91.89%
STD	30.11%	52.11%	76.78%	85.89%	88.44%	88.00%	91.22%	91.78%
Peak	30.67%	41.89%	63.67%	73.22%	76.89%	79.00%	81.56%	80.44%
RMSEE	23.89%	41.78%	46.22%	52.33%	53.78%	55.44%	56.78%	52.78%
WFE	1.11%	7.22%	7.22%	20.22%	24.11%	27.22%	39.78%	46.44%
Kurtosis	2.67%	9.67%	20.78%	23.44%	12.33%	12.11%	29.56%	31.00%
Skewness	1.78%	6.56%	15.11%	20.11%	23.11%	22.89%	22.11%	23.78%
CRF	0.44%	2.89%	1.56%	3.56%	10.22%	10.67%	10.56%	14.22%
IMF	1.89%	6.89%	8.89%	21.33%	12.78%	12.22%	10.33%	12.22%
All	48.33%	73.00%	86.11%	92.33%	94.33%	95.78%	97.22%	97.89%

**Table 5:** Multifeature fusion performed using 2–9 different features in turn, while the fusion order was changed at the same time. For example, the first feature value in the third row of the table below is STD, and the subsequent fusion order is RMS, Peak, RMSEE, WFE, etc.

Eigenvalue	RMS	STD	Peak	RMSEE	WFE	Kurtosis	Skewness	CRF	IMF
RMS		79.67%	79.00%	80.33%	82.78%	83.89%	85.11%	86.33%	86.11%
STD	79.67%		79.00%	80.33%	82.78%	83.89%	84.67%	86.33%	86.00%
Peak	79.22%	79.44%		80.00%	82.89%	83.89%	84.56%	85.67%	85.11%
RMSEE	79.67%	81.33%	79.78%		82.89%	83.78%	85.33%	86.00%	86.22%
WFE	81.78%	82.33%	83.11%	83.22%		84.00%	84.33%	85.44%	86.00%
Kurtosis	82.44%	84.11%	82.89%	83.00%	84.00%		83.89%	85.00%	85.56%
Skewness	82.22%	82.56%	83.22%	83.44%	84.44%	84.44%		85.44%	85.00%
CRF	81.33%	80.44%	81.11%	81.44%	82.33%	84.89%	85.33%		85.00%
IMF	82.33%	83.67%	82.11%	82.44%	83.00%	83.89%	84.78%	86.22%	

**Table 6:** Optimal combination of features and fault prediction accuracy for different sliding windows, where “All” is the corresponding multifeature combination in Table 2, specifically {RMS, STD, Peak, RMSEE, WFE, Kurtosis, Skewness, CRF, IMF}.

Sliding Window Size	Optimal Feature Combination	Accuracy	
		All	Optimal Combination
120	{Kurtosis,RMS,STD,Peak,RMSEE,WFE,Skewness,CRF}	48.33%	50.44%
240	{RMS,STD,Peak,RMSEE,WFE,Kurtosis,Skewness,CRF,IMF}	73.00%	73.00%
360	{RMS,STD,Peak,RMSEE,WFE,Kurtosis,Skewness,CRF}	86.11%	86.33%
480	{WFE,RMS,STD,Peak,RMSEE,Kurtosis,Skewness,CRF,IMF}	92.33%	93.00%
600	{RMS, STD,Peak,RMSEE,WFE,Kurtosis,Skewness,CRF,IMF}	94.33%	94.33%
720	{IMF,RMS,STD,Peak,RMSEE,WFE,Kurtosis,Skewness}	95.78%	96.44%
840	{Skewness,RMS,STD,Peak,RMSEE,WFE,Kurtosis,CRF,IMF}	97.22%	97.67%
960	{RMS,STD,Peak,RMSEE,WFE,Kurtosis,Skewness,CRF,IMF}	97.89%	97.89%

**Table 7:** Structure of the ANN and the variation range of relevant parameters.

Parameter	Range Interval/Value
Number of hidden layers	1
Number of hidden layer units	[10, 100]
Learning rate	[0.0001, 0.1]
Momentum parameter	[0.001, 0.999]
RMSprop parameter	[0.001, 0.999]

**Table 8:** ANN parameters, loss value, and prediction accuracy obtained using the PSO-ANN model for multifeature fusion fault prediction.

Number of Particles	Learning Rate	Momentum Parameter	RMSprop Parameter	Number of Hidden Layer Neurons	Loss Value	Accuracy
10	0.021404	0.999	0.999	100	0.372830	89.22%
20	0.007614	0.609325	0.658986	58	0.479214	89.44%
30	0.006649	0.573852	0.966601	81	0.464076	89.89%
40	0.008156	0.467269	0.989776	77	0.467528	89.22%
50	0.014367	0.998993	0.999	90	0.347928	90.11%
60	0.010740	0.999	0.999	81	0.349434	89.67%

**Table 9:** Accuracy of fault prediction based on the PSO-ANN model.

Eigenvalue	Sliding Window Size							
	120	240	360	480	600	720	840	960
RMS	40.00%	58.89%	78.33%	87.11%	89.22%	88.78%	91.56%	92.00%
STD	41.22%	64.22%	78.00%	86.44%	89.22%	88.56%	91.78%	92.11%
Peak	42.67%	58.11%	68.00%	76.33%	77.44%	81.11%	82.22%	81.78%
RMSEE	33.00%	47.67%	59.44%	62.44%	70.89%	70.89%	72.44%	75.33%
WFE	7.33%	10.67%	20.56%	30.33%	32.33%	42.56%	47.44%	49.89%
Kurtosis	5.89%	11.44%	24.33%	25.67%	21.00%	23.11%	41.44%	47.00%
Skewness	3.22%	11.44%	19.78%	20.44%	23.56%	24.11%	23.56%	30.22%
CRF	1.11%	4.89%	7.89%	20.11%	21.78%	11.44%	12.56%	15.67%
IMF	4.11%	10.33%	20.00%	23.89%	14.78%	14.22%	34.78%	32.56%
All	54.67%	78.44%	90.11%	93.11%	96.22%	97.22%	97.89%	98.67%

**Table 10:** Prediction results of test data from multiple PSO-ANNs trained by different single features.

PSO-ANN Model	Fault Type					
	Normal State	Inner Race Fault	Rolling Element Fault	Outer Race Orthogonal@3:00 Fault	Outer Race Centered@6:00 Fault	Outer Race Opposite@12:00 Fault
STD	0	0.2979	0.0053	0.1500	0.2961	0.2507
Peak	0	0.267	0.0608	0.1630	0.2214	0.2878
RMSEE	0	0.2763	0.0846	0.1170	0.2759	0.2462
Skewness	0.0926	0.0674	0.1257	0.2928	0.227	0.1945

**Table 11:** Parameter values obtained by preprocessing the data in Table 9 according to Algorithm 2.

Parameter Name	PSO-ANN Trained by a Single Feature			
	STD	Peak	RMSEE	Skewness
PRE	0.2941	0.2623	0.2672	0.1789
CRD	0.2934	0.2616	0.2665	0.1785
MUN	7.3255	8.8422	8.9058	11.2462
MCRD	2.1493	2.3132	2.3734	2.0073
NMCRD	0.243	0.2616	0.2684	0.227

**Table 12:** Results of decision-level fusion using the DS evidence theory. The number of fusions was 0, which indicates that the results were calculated by Equation (12).

Fusion Times of DS	Fault Type					
	Normal State	Inner Race Fault	Rolling Element Fault	Outer Race Orthogonal@3:00 Fault	Outer Race Centered@6:00 Fault	Outer Race Opposite@12:00 Fault
0	0.021	0.2317	0.0684	0.1769	0.2555	0.2465
1	0.002	0.2484	0.0217	0.1448	0.302	0.2811
2	0.0001	0.249	0.0065	0.1109	0.3338	0.2997
3	0	0.2435	0.0019	0.0828	0.36	0.3118

**Table 13:** Fault prediction accuracy of decision-level fusion using multiple PSO-ANN models trained with different single features combined with different DS evidence theory.

Method	Sliding Window Size							
	120	240	360	480	600	720	840	960
Basic DS	67.89%	82.00%	92.44%	95.89%	97.44%	97.89%	98.89%	98.89%
Literature [30]	67.56%	82.56%	92.44%	96.22%	97.44%	97.89%	98.89%	98.78%
Literature [31]	68.44%	81.78%	92.33%	96.22%	97.44%	98.00%	98.78%	98.89%
Literature [32]	68.22%	81.67%	92.33%	96.22%	97.33%	98.00%	98.78%	98.89%
We Proposed	68.33%	82.67%	92.44%	96.44%	97.44%	98.22%	99.00%	99.00%

**Table 14:** Fault prediction accuracy of various models.

Model	Sliding Window Size							
	120	240	360	480	600	720	840	960
KNN	57.78%	74.45%	84.33%	90.11%	93.11%	94.67%	95.44%	96.44%
Decision tree	57.22%	75.44%	86.89%	91.44%	94.00%	95.67%	97.11%	98.22%
Random forest	61.89%	78.00%	89.33%	94.00%	96.44%	97.33%	97.78%	98.44%
Naive Bayes	62.11%	76.33%	83.67%	90.56%	93.78%	95.00%	97.44%	98.11%
ANN	50.44%	73.00%	86.33%	93.00%	94.33%	96.44%	97.67%	97.89%
SVM	63.67%	78.89%	88.00%	92.67%	95.11%	96.78%	97.78%	98.00%
LSTM	57.89%	72.89%	80.11%	84.22%	88.33%	91.56%	93.00%	96.11%
PSO-ANN	54.67%	78.44%	90.11%	93.11%	96.22%	97.22%	97.89%	98.67%
PSO-ANN-DS	68.33%	82.67%	92.44%	96.44%	97.44%	98.22%	99.00%	99.00%
